# Editorial: Childhood diabetes in low- and middle-income countries: progress, challenges, and actions needed, volume II

**DOI:** 10.3389/fendo.2023.1304716

**Published:** 2023-10-19

**Authors:** Mitchell E. Geffner

**Affiliations:** Division of Endocrinology, Diabetes, and Metabolism, The Saban Research Institute, Children’s Hospital Los Angeles, Keck School of Medicine of the University of the Southern California, Los Angeles, CA, United States

**Keywords:** autoantibodies, celiac disease, circular ribonucleic acids, diabetes, glucagon-like peptide-1 receptor agonists, low-and middle-income countries

According to the most recent projections, there were 425 million cases (~6% of the world’s population) of diabetes worldwide in 2017 (1). This prevalence has been rising more rapidly in low- and middle-income than in high-income countries (2). Approximately 80% of adults with diabetes worldwide live in low- and middle-income countries (3). It is estimated that globally, in 2017, there were 9 million people with type 1 diabetes (T1D) (4). Of this number, 2.6 million people with type 1 diabetes (29%) live in lower-middle or low-income countries (5). In many low- and middle-income countries, the true burden of T1D is unknown and health systems are poorly equipped to address this complex chronic disease, including poor availability and affordability of insulin (6).

The World Health Organization (WHO) promotes the adoption of effective measures for the surveillance, prevention, and control of diabetes and its complications, especially in low- and middle-income countries. As such, the WHO offers scientific guidelines for the prevention of multiple major non-communicable diseases, including diabetes; develops standards for the diagnosis of diabetes and the care of affected patients; builds awareness regarding the global epidemic of diabetes, including the celebration of World Diabetes Day (November 14); and undertakes surveillance of the prevalence of diabetes and its risk factors. This effort has included the unveiling – in April 2021 - of the Global Diabetes Compact, a global initiative with the goal of maintaining sustained improvements in diabetes prevention and care, especially in low- and middle-income countries (7). Additionally, in May 2021, the 75^th^ World Health Assembly agreed upon a resolution to promote the prevention and control of diabetes which - in May 2022 – included the endorsement of multiple targets for global diabetes coverage and treatment to be achieved by 2030.

The objective of the second Research Topic on *“Childhood diabetes in low- and middle-income countries: progress, challenges, and actions needed, volume II”* is to continue to gather original research articles involving recent advances related to T1D in low- and middle-income countries. This continuing Research Topic consists of five original articles. The messages of these articles are summarized below.

Su et al. from Tongji Medical College, Huazhong University of Science & Technology, Wuhan, China, using data from China’s annual report on national health statistics and the Chinese Health Statistics Yearbook, have analyzed the time trends of mortality rates among patients with diabetes in the rural and urban population in China between 1987 and 2019. They report that the age-standardized mortality rate of diabetes is on the rise in China especially in rural areas (increasing by about 39% in urban areas and 255% in rural areas over the entire study period).

To better understand the role of autoimmune injury as the basis for the increasing frequency of celiac disease autoantibody (CDAb) seropositivity in patients with T1D, Zhang et al. investigated the role of circular ribonucleic acids (circRNAs), which are known to participate in autoimmune diseases. Among 80 patients diagnosed with T1D who were screened for CDAbs and CD-predisposing genes, circRNAs were analyzed in peripheral blood mononuclear cells (PBMCs) from 47 patients. The Gene Expression Omnibus (GEO) database was searched for candidate circRNAs in related studies on T1D PBMCs. The investigators found a high percentage (35.0%) of patients were positive for CDAbs. The profile of candidate circRNAs in children with T1D with CDAbs in this study was different from that in previous reports in patients with T1D from the GEO database. The findings also showed that *hsa_circ_0004564* and its parental gene, *RAPH1*, may be new targets for studying immune mechanisms in children with T1D and CD. Most importantly, this study highlights the importance of screening for CD in Chinese children with T1D, given the high prevalence of CDAb positivity and CD-predisposing genes that was detected.

In a cohort of children with T1D and their siblings in Sri Lanka, Atapattu et al. report the prevalence of diabetes- and thyroid-related autoantibodies which has not been previously well-studied in this country. Using 3-Screen ICA™ (3-Screen) and individual ELISA assays to measure antibodies to glutamic acid decarboxylase (GAD), insulinoma-associated antigen-2 (IA-2A) and zinc transporter 8 (ZnT8A), along with insulin (IAA), thyroid peroxidase (TPOA), and thyroglobulin (TgA), the investigators found that, among T1D children: (i) 79.1% were 3-Screen positive and all 3-Screen positives had individual rates of 74% for GAD, 31% for IA-2A, and 39% for ZnT8A, and were younger than 3-Screen negative subjects; (ii) 45% had multiple autoantibodies; and (iii) TPOA and TgA prevalence was higher in T1D children compared to unaffected siblings. Among unaffected siblings, 6.3% were 3-Screen positive and 2.4% were IA-2A-positive, while four subjects had two diabetes-related autoantibodies, one of whom developed dysglycemia during follow-up. These findings resemble those in other populations.

Because many patients with T1D are most probably not diagnosed at all in a number of the low- and middle-income countries, which may contribute to their apparent low incidence, Ludvigsson et al. sought to accurately determine its incidence (and clinical presentation and course) in youth/young people in Tanzania, a country with low income and poor resources, using casebooks and statistics at several Tanzanian hospitals treating young people with T1D, and collections of information from different organizations such as the Tanzanian Diabetes Association, Life for a Child, Changing Diabetes in Children, and World Diabetes Foundation. The incidence in several geographical areas was low, but accuracy of incident cases was affected by missing data at studied clinics therefore probably underestimating true incidence. Most patients presented with typical symptoms and signs of T1D, including a high proportion with ketoacidosis, although less often than previously (~90% down to ~40% in recent decades). After diagnosis, many patients have poor blood glucose control, and complications may be present after a relatively short duration of disease. Over the years, resources have improved, awareness has increased, and well-staffed diabetes clinics have evolved, but there is still much work to be done.

With the increasing usage of glucagon-like peptide-1 receptor agonists (GLP-1RAs) to treat obesity and type 2 diabetes mellitus (T2DM) in adults and adolescents in the west, Yan et al. sought to explore the frequency and appropriateness of their use in children and adolescents in China. The latter was determined from the indications approved by China National Medical Products Administration (NMPA), as well as multiple other international regulatory agencies and published randomized controlled trials. There were found 234 prescriptions from 46 hospitals, with the median age of the patients being 17 years. The majority were diagnosed with overweight/obesity (44%) or prediabetes/diabetes (46%). There were 88 monotherapy prescriptions, while combination therapy included GLP-1RAs + metformin (39%) and GLP-1RAs + orlistat (12%). The number of prescriptions for overweight/obesity increased from 27% in 2016 to 54% in 2021, while prescriptions for prediabetes/diabetes actually decreased from 55% to 42%. The prescriptions were categorized as appropriate versus questionable by diagnosis, and the potentially questionable prescription use was statistically related to age, department visited, and the occurrence of any hospitalization. From their results, the authors remind us that it is crucial to demand strong and sustained efforts to enhance the awareness (in China) of the utilization, benefits, and safety of GLP-1RAs in children and adolescents.

In summary, the second volume of “*Childhood diabetes in low- and middle-income countries: progress, challenges, and actions needed*” has provided new information re prevalence, diagnosis, co-morbidities, and treatment of diabetes. The continued conduct of these types of research in low- and middle-income countries is vital in order to improve all aspects of diabetes care in under-resourced communities around the world.

## Author contributions

MG: Writing – original draft.
